# Corticospinal tract integrity and motor function following neonatal stroke: a case study

**DOI:** 10.1186/1471-2377-12-53

**Published:** 2012-07-09

**Authors:** Anne L Gordon, Amanda Wood, Jacques Donald Tournier, Rod W Hunt

**Affiliations:** 1 Evelina Children’s Hospital, Guys’ & St Thomas’ Hospital NHS Trust, London, UK; 2 Murdoch Children’s Research Institute, Melbourne, Australia; 3 Department of Paediatrics, University of Melbourne, Melbourne, Australia; 4 School of Psychology, University of Birmingham, Edgbaston, UK; 5 Brain Research Institute, Florey Neuroscience Institutes (Austin), Melbourne, Australia; 6 Department of Medicine, Austin Health and Northern Health, University of Melbourne, Melbourne, Victoria, Australia; 7 Department of Neonatal Medicine, The Royal Children’s Hospital, Flemington Road, Parkville, Victoria, 3052, Australia

**Keywords:** Stroke, Children, Plasticity, Functional imaging, Motor outcome

## Abstract

**Background:**

New MRI techniques enable visualisation of corticospinal tracts and cortical motor activity. The objective of this case study was to describe the magnetic resonance evidence of corticospinal pathway reorganisation following neonatal stroke.

**Case presentation:**

An 11 year old boy with a neonatal right middle cerebral artery territory ischaemic stroke was studied. Functional MRI was undertaken with a whole hand squeezing task, comparing areas of cortical activation between hands. White matter tracts, seeded from the area of peak activation in the cortex, were visualised using a diffusion weighted imaging probabilistic tractography method. Standardised evaluations of unilateral and bilateral motor function were undertaken. Clinically, the child presented with a left hemiparesis. Functional MRI demonstrated that movement of the hemiparetic hand resulted in activation in the ipsi-lesional (right) hemisphere only. Diffusion tractography revealed pathways in the right (lesioned) hemisphere tracked perilesionally to the cortical area identified by functional MRI.

**Conclusion:**

Our case demonstrates that neonatal stroke is associated with maintenance of organization of corticospinal pathways sufficient to maintain some degree of hand function in the affected hemisphere. Functional MRI and diffusion weighted imaging tractography may inform our understanding of recovery, organisation and reorganisation and have the potential to monitor responses to intervention following neonatal stroke.

## Background

Neonatal arterial ischaemic stroke (AIS) is an important cause of cerebral palsy. The middle cerebral artery is the most common vascular territory affected. Functional consequences are diverse, and may emerge over time. Hemiparesis is less common in neonatal onset stroke when compared with childhood onset [1]. It has been postulated that the developmental stage of the brain at the time of injury may influence recovery from brain injury in children. A number of mechanisms may be responsible for cerebral recovery including formation of new synaptic connections, change of function of neurones in response to injury, and utilisation of pathways contralateral to the injury site [2].

Magnetic resonance imaging (MRI) techniques enable visualisation of corticospinal tracts (using diffusion-weighted imaging (DWI)) and cortical motor activity (using functional MRI (fMRI)). This information has the potential to be used to explore mechanisms of recovery and reorganisation and to measure response to interventions aimed at improving outcome. The aim of this study was to investigate whether there was evidence of relocation of cortical motor activity and reorganisation of motor pathways in a school- aged child who had a unilateral perinatal AIS affecting the motor cortex with residual hemiparesis.

## Case presentation

### Method

The subject, an 11 year old boy, sustained an AIS affecting the right middle cerebral artery territory diagnosed radiologically during the perinatal period. Perinatal MR imaging had previously been obtained on a 1.5-Tesla General Electric LX Echospeed system (version 9.0). Transverse T1 fast spin echo (TR/TE 1400/10, ETL 2, BW +/− 20 kHz, FOV 20 cm, slice thickness 3.5 mm; 256 × 224 matrix, 2 averages) and transverse fast spin echo T2 (TR/TE 3600/16, ETL 18, BW +/− 20.83 kHz, FOV 18 cm, slice thickness 3.0 mm, 256 × 224 matrix, 3 averages), epi-DWI (3-directions, b = 1 ms/μm^2^, TR/TE 10000/104, BW +/− 100 kHz, FOV 25 cm, slice thickness 4.0 mm,192 × 128 matrix, 2 averages) were obtained.). Ethics approval for the current study was obtained. Clinical evaluation was undertaken by an experienced paediatric occupational therapist (AG) and included the Bruininks-Oseretsky Test of Motor Proficiency 2 (BOT2) [3], Assisting Hand Assessment (v4.1) (AHA) [4] and Pediatric Stroke Outcome Measure (PSOM) [5]. The BOT2 provides standard scores for overall motor function based on age-matched peers (mean 100, sd 15). The AHA enables calculation of the effectiveness of use of the assisting hand and arm in bimanual activities and scores are expressed as percentage score out of maximum of 100. The PSOM is a standardised neurological examination expressing neurological impairment across domains as a total on a scale of 1–10. Motor abilities were further classified using the Manual Ability Classification System (MACS) [6] and Gross Motor Functional Classification System (Expanded & Revised) (GMFCS) [7] both 5 point scales of motor function. Motor outcome measures were scored in relation to normative data (BOT2) or reported descriptively.

The imaging protocol undertaken included T1-weighted imaging of the whole brain (3D high resolution (3D MPRAGE)) and T2-weighted imaging (turbo spin echo and T2 FLAIR imaging). DWI was obtained with 60 directions (b-value = 3000 s/mm^2^, 2.3 × 2.3 × 2.3 mm^3^ voxel size). fMRI consisted of multi-slice 2D echo planar imaging, with a whole-hand ball squeezing task in a block design with five epochs. Five cycles of task (hand squeeze) and rest (no movement) were performed per epoch. fMRI data were processed using Statistical Parametric Mapping (v5) [8]. The realignment was processed using FLIRT, and mean fMRI co-registered to the b = 0 s/mm^2^ image using the default metric. Peak activation coordinates were transformed to the DWI space by applying the transform to the native coordinates. Data was smoothed to 11 mm, and statistics were family-wise error corrected at p < 0.05. Areas of peak activation for each hand were identified by coordinates, which were then used to seed diffusion weighted tractography (DWT) of the corticospinal tracts, comparing left and right hands. The DWT was undertaken using a probabilistic streamlines approach capable of tracking through crossing fibre regions [9-11] as implemented in the MRtrix software package (http://www.brain.org.au/software). Tracking was from a 3 mm radius sphere centered on peak fMRI activation (more on that below) for each side, down to a 10 mm radius sphere centered on the medulla oblongata, located immediately inferior to the pons on the sagittal image. The tracks generated were then resampled at 2 mm intervals in the inferior-superior direction.

### Results

The subject presented with a MACS level 2 and GMFCS level 2 left hemiparesis, and a PSOM score of 1. He was able to achieve an inferior pincer grip but not reliably, with his left (affected) hand. The left arm and hand were used to assist in bilateral activities by holding with a restricted repertoire of movements. Consistent with this functional picture, his AHA score was 65 % and BOT2 total composite standard score was 33 (below average).

**Figure 1:**
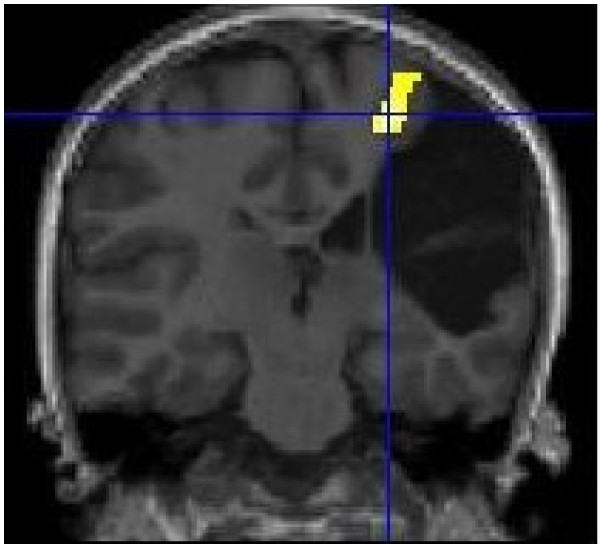
**Motor fMRI Findings.** Coronal view. fMRI activation in R cortical region with left (affected) hand motor task. (R side of brain appears on L side of image).

**Figure 2:**
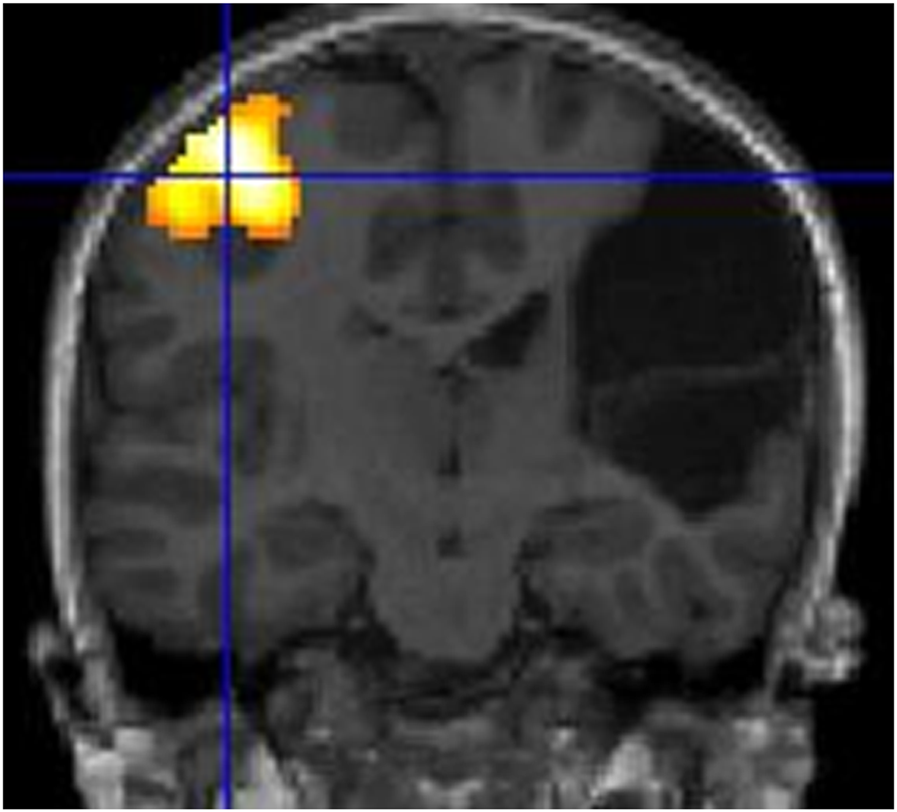
**Motor fMRI Findings.** Coronal view. fMRI activation in L cortical region with right hand motor task (R side of brain appears on L side of image).

**Figure 3:**
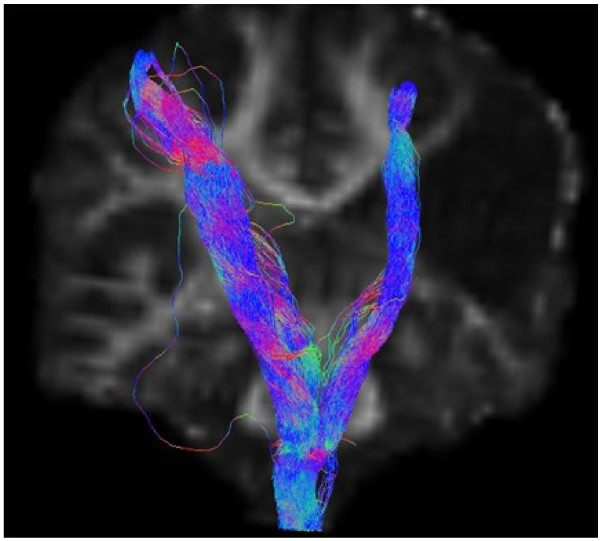
**DWI Tractography Findings.** DWI tractography seeded from coordinates of maximal activation on fMRI to the brainstem (R side of brain appears on L side of image).

**Figure 4:**
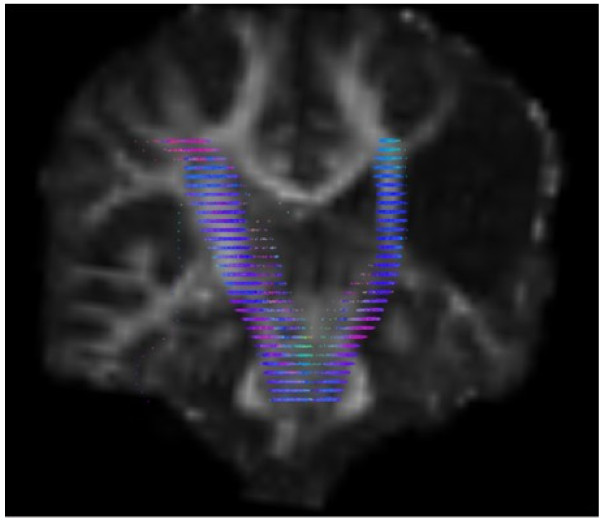
**DWI Tractography Findings.** Fractional anisotropy values were calculated at 2 mm intervals, resampled from the pons to the centrum semiovale. (R side of brain appears on L side of image).

**Figure 5:**
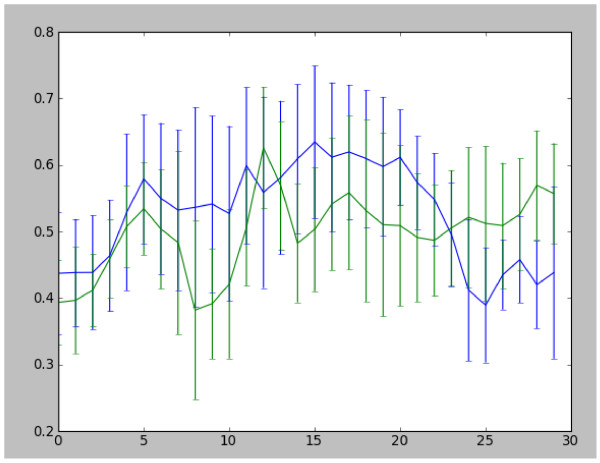
**FA Values Along Corticospinal Tracts.** FA values at 2 mm intervals (displayed in Figure 4) shown numerically, showing reduced FA in the cortico-spinal tracts in inferior regions on lesioned right side compared to left. X axis: FA values from inferior (lower end of axis) to superior structures (upper end). Right hemisphere (lesioned side) tracts green, left hemisphere tracts blue.

On structural MRI, the site of the brain injury was identified in the right perisylvian region with cystic leukoencephalomalacia involving posterior right frontal, right parietal pre- and post-central gyri and superior temporal lobe. Comparison of cortical activation between left and right hemispheres using fMRI demonstrated that movement of the affected left hand resulted in activation in the ipsi-lesional hemisphere only, anterior to the lesion within the motor cortex. In comparison, movement of the right hand resulted in expected activation of the motor cortex. DWT revealed pathways in the right (lesioned) hemisphere tracked perilesionally to the cortical area identified by fMRI. The right hand motor task resulted in expected and more robust motor cortical activation with the expected contralateral hemisphere and corticospinal tracts when compared to left (see Figures 1, 2). DWT was seeded from areas of maximal fMRI activation to the pons (Figure 3). Sampling of FA values sequentially along the corticospinal tracts (Figures 4, 5) shows reduced FA in the ipsilesional tracts when compared to the contralesional side.

## Conclusion

The DWT results described in this case show that the region identified by fMRI as supporting hemiparetic hand use has the necessary white matter connections to perform this function. The underlying white matter may not be sufficiently preserved to allow a full recovery of function, but partial recovery is evident. The reduced FA within the ipsilesional cortico-spinal tract compared to the contralesional side may be explained by a number of factors. These include absence of myelin; compromised axonal membranes; reduced axonal density; and increased extra-cellular water [12]. In this case however, it is unlikely given the child’s medical history that the observed FA changes are due to demyelination or compromised axonal membranes. Given the nature of the brain insult, it may however be expected that there would be loss of axonal density, and corresponding increase in extra-cellular water, due to loss of some projections to/from the site of injury.

Our findings suggest that chronic functional motor deficits following neonatal AIS may be associated with limited preservation of organisation to ipsilesional regions of the cortex, and there was reduced pathway density in regions of the tracts distal to the site of injury. The underlying mechanisms for these processes remain unknown. Wallerian degeneration of the descending corticospinal tracts has been associated with poor motor outcome in both acute and chronic paediatric stroke [13,14].

In this case, despite unilateral damage to the motor cortex, the child developed useful functional ability of the impaired upper limb, as a non-dominant limb. DWT suggests intact perilesional motor pathways were associated with the use of the child’s impaired hand. fMRI and DWI of structures associated with motor outcome may improve our ability to predict outcome following paediatric stroke. In paediatric clinical practice a number of approaches have shown potential to improve motor function including transcranial magnetic stimulation [15], modified constraint induced movement therapy, and bimanual training [16]. It is possible that activity-dependent synaptic plasticity and reorganisation of motor and sensory maps is influenced by lesion, age and intervention characteristics. Prospective magnetic resonance studies imaging motor tracts longitudinally in relation to functional recovery may assist in identifying targets for motor rehabilitation intervention. Future studies are indicated to explore the impact of intervention on axonal density in the corticospinal tracts and functional outcome.

## Summary

DWT enables visualisation and quantification of white matter tracts and can inform our understanding of mechanisms of recovery in early acquired damage to the developing brain. Further studies are needed to explore the relationship between white matter tracts (as characterised using DWT) and the degree of motor recovery. This has the potential to underpin rehabilitation interventions aimed at improving motor function following stroke in infancy.

## Consent

Written informed consent was obtained from the patient’s parents for publication of this Case report and any accompanying images. A copy of the written consent is available for review by the Series Editor of this journal.

## Abbreviations

MRI, Magnetic resonance imaging; fMRI, Functional MRI; DWT, Diffusion weighted tractography; DWI, Diffusion-weighted imaging, AIS, Arterial ischaemic stroke (AIS, BOT2, Bruininks-Oseretsky Test of Motor Proficiency 2; AHA, Assisting Hand Assessment v4.1, PSOM, Pediatric Stroke Outcome Measure, MACS, Manual Ability Classification System; GMFCS, Gross Motor Functional Classification System (Expanded & Revised).

## Competing interests

The authors declare they have no competing interests.

## Authors’ contributions

AG conceived of the study, undertook the clinical examinations, participated in the imaging data acquisition and drafted the manuscript; JDT participated in the design of the study, performed diffusion weighted imaging analysis and data interpretation and helped draft the manuscript; AW participated in the imaging protocol design, undertook functional imaging analysis and interpretation and helped draft the manuscript; RH conceived of the study, participated in its design and coordination, and drafting of the manuscript. All authors read and approved the final manuscript.
